# Predictive Factors of Tolerance in Office Hysteroscopy – a 3-Year Analysis from a Tertiary Center

**DOI:** 10.1055/s-0043-1764361

**Published:** 2023-03-06

**Authors:** Ana Carolina Coimbra, Vera Falcão, Pedro Pinto, João Cavaco-Gomes, Ana Sofia Fernandes, Margarida Martinho

**Affiliations:** 1 Department of Gynecology, Centro Universitário Hopitalar de São João, Porto, Portugal; 2 Department of Gynecology, Centro Hospitalar do Médio Ave, Vila Nova de Famalicão, Portugal; 3 Department of Anatomy, Faculdade de Medicina da Universidade do Porto (FMUP), Porto, Portugal

**Keywords:** office hysteroscopy, tolerance, pain, risk factor, outpatient

## Abstract

**Objective** Pain is the primary limitation to performing hysteroscopy. We aimed to evaluate the predictive factors of low tolerance to office hysteroscopic procedures.

**Methods** Retrospective cohort study of the patients who underwent office hysteroscopy from January 2018 to December 2020 at a tertiary care center. Pain tolerance to office-based hysteroscopy was subjectively assessed by the operator as *terrible* , *poor* , *moderate* , *good* , or *excellent* . Categorical variables were compared with the use of the Chi-squared test; an independent-samples t-test was conducted to compare continuous variables. Logistic regression was performed to determine the main factors associated with low procedure tolerance.

**Results** A total of 1,418 office hysteroscopies were performed. The mean age of the patients was 53 ± 13.8 years; 50.8% of women were menopausal, 17.8% were nulliparous, and 68.7% had a previous vaginal delivery. A total of 42.6% of women were submitted to an operative hysteroscopy. Tolerance was categorized as *terrible* or *poor* in 14.9% of hysteroscopies and *moderate* , *good,* or *excellent* in 85.1%. A *terrible* or *poor* tolerance was more frequently reported in menopausal women (18.1% vs. 11.7% in premenopausal women, *p* = 0.001) and women with no previous vaginal delivery (18.8% vs. 12.9% in women with at least one vaginal birth, *p* = 0.007). Low tolerance led more often to scheduling a second hysteroscopic procedure under anesthesia (56.4% vs. 17.5% in *reasonable* -to- *excellent* tolerance, *p* < 0.0005).

**Conclusion** Office hysteroscopy was a well-tolerated procedure in our experience, but menopause and lack of previous vaginal delivery were associated with low tolerance. These patients are more likely to benefit from pain relief measures during office hysteroscopy.

## Introduction

Hysteroscopy has emerged as the gold-standard for the investigation and treatment of uterine pathology. 1 It is considered the most accurate method in the study of a wide spectrum of gynecological conditions, such as abnormal uterine bleeding, postmenopausal bleeding, and infertility. 1 2 3 Originally, hysteroscopic procedures required wider diameter sheaths, speculum and tenaculum placement, cervical dilators, and carbon dioxide for uterine distention, which were largely responsible for eliciting pain and vasovagal reactions. 4 Due to technological advances over the last 20 years, progressive miniaturization of hysteroscopic instruments and technique improvements have increased the acceptability of performing hysteroscopy in a more cost-effective outpatient setting, avoiding the operating room and the risks of undergoing anesthesia. 2 4 5 6

This technique is no longer a simple diagnostic tool as it allows a sequential approach to most intrauterine pathologies (known as “see and treat”), in which diagnosis is immediately followed by treatment. 1 4 7 An increasing variety of procedures are performed in an office-based setting, such as polypectomy, myomectomy, adhesiolysis, metroplasty, directed biopsy, and removal of retained products of conception or embedded intrauterine devices. 1 8 Ambulatory gynecology has substantial health and economic benefits and office-based hysteroscopy has gradually become a common practice, allowing for patients to resume their normal daily activity shortly after its completion, with low complication rates and faster recovery from increasingly less painful procedures. 1 9 Histeroscopy is generally regarded as a well-tolerated technique, and several studies suggest that only a few selected patients have the need for analgesia, namely nulliparous and menopausal women, women with previous cesarean section, history of chronic pelvic pain or anxiety. 7 10 However, pain and low pain tolerance are still the primary limitations to performing hysteroscopy or to complete the procedure in an office setting. 2 3 6 11

Numerous pharmacological and non-pharmacological treatments for pain relief have been suggested, with conflicting results. 5 8 Thus, there are no specific guidelines regarding their use for office-based hysteroscopy. 12 As such, clarifying the main factors associated with low tolerance to the procedure would enable us to anticipate the need for pain relief treatment, adapting to each patient individually. In this study, we aimed to evaluate predictive factors of low tolerance to office hysteroscopic procedures.

## Methods

We carried out a retrospective cohort study by reviewing the electronic medical records of all the patients who underwent office hysteroscopy from January 2018 to December 2020 at a tertiary care center. The study protocol was approved by the institutional ethics committee. Data obtained included patient demographics, such as age, parity, and number of vaginal and cesarean deliveries. Also, menopausal status was assessed as well as whether the patient was on hormone therapy. We collected information on the main indication for performing the procedure and if cervical priming with misoprostol prior to the scheduled outpatient intervention was prescribed (400 micrograms single sublingual dose, two hours before the examination, according to our department protocol). A rigid hysteroscope with an optic of 30 degrees was used. Hysteroscopic sheath diameter varied from 3.5 to 5 mm, and the uterine cavity distension medium was saline solution. Hysteroscopies were either performed or supervised by an experienced hysteroscopist. The need for paracervical block with a solution of 2% lidocaine was noted. We documented intraoperative findings, namely intra-cavitary pathology, and which (if any) operative interventions were carried out. Pain tolerance to office-based hysteroscopy was subjectively assessed by the operator and classified as *terrible* , *poor* , *moderate* , *good* , or *excellent* . For the statistical analysis, patients with terrible and *poor* tolerance were rated as having low tolerance, while if procedure tolerance was *moderate* , *good* , or *excellent* , it was considered well tolerated. We recorded the occurrence of vasovagal reaction and if a second appointment was needed to complete the procedure.

## Statistical analysis

Categorical variables are presented as frequencies and percentages, and continuous variables as means and standard deviations. Normal distribution was checked using skewness and kurtosis. Categorical variables were compared with the use of the Chi-squared test, and an independent-samples t-test was conducted to compare continuous variables. Multivariate analysis through logistic regression was performed to determine the main factors associated with low procedure tolerance. All reported *p* -values are two-tailed, with a *p* -value of 0.05 indicating statistical significance. Analysis was performed with the use of IBM SPSS Statistics for Windows, version 23.0 (IBM Corp., Armonk, NY, USA).

## Results

During the 3-year study period, 1,418 office hysteroscopies were performed. Regarding epidemiological data, patients had a mean age of 53 ± 13.8 years, 695 were postmenopausal (49%) and among these, 62 were on menopausal hormone therapy. A total of 107 patients were under treatment with tamoxifen (7.5%) ( Table 1 ). Regarding parity, 16.1% of patients were nulliparous, 61.4% had at least 1 vaginal delivery, and 23.8% had at least 1 cesarean section. Sonographic suspicion of endometrial polyp was the main indication for hysteroscopic evaluation, comprising 41.5% of cases, followed by asymptomatic endometrial thickening (27.8%), abnormal uterine bleeding/postmenopausal bleeding (8.9%), retained intrauterine device (5.2%), and infertility (4.9%). Cervical priming with misoprostol was registered in 16.9% of cases (80.6% of them in menopausal women), and paracervical block was performed in 2% of the procedures. Vaginoscopic technique was used in all hysteroscopies, with no need for insertion of speculum or tenaculum, aside from the cases with paracervical block.

**Table 1:** Clinical and procedure characteristics of the patients submitted to office hysteroscopy

Patient/procedure characteristics	N (%)
Menopause	695 (49.0)
Menopausal hormone therapy	62 (4.4)
Tamoxifen	107 (7.5)
Nulliparous	229 (16.1)
Previous vaginal delivery	871 (61.4)
Previous cesarean section	338 (23.8)
Cervical priming with misoprostol	240 (16.9)
Paracervical block	28 (2.0)
Operative hysteroscopy	604 (42.6)

**Fig. 1:**
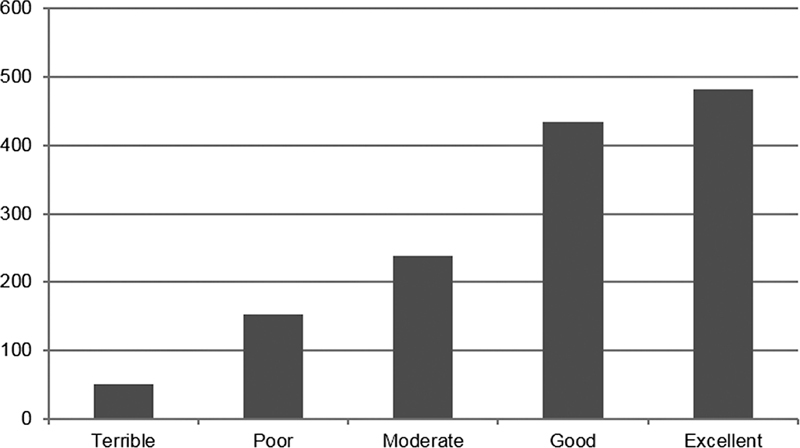
Patient distribution according to classification of pain tolerance to office hysteroscopy.

Intracavitary pathology was documented in 75% of cases. Endometrial polyp was the most frequent finding, seen in 625 patients (44.1% of abnormal findings), followed by endometrial hypertrophy (9.1%), submucosal myoma (8.5%), and endometrial synechiae (6.5%). In 814 women (57.4%), a merely diagnostic hysteroscopy was conducted; therefore, a smaller diameter sheath was used, while 604 women were submitted to an operative hysteroscopy (5 mm diameter sheath). Polipectomy was done in 360 patients (25.5%). The other most common operative procedures were directed endometrial biopsy in 94 women (6.6%) and removal of retained intrauterine device in 72 (5.1%). As for reported complications, vasovagal reaction was documented in 35 women (2.5%) and a false passage in 4 cases. Tolerance to outpatient hysteroscopy was immediately assessed by the operator following completion of the procedure. Tolerance was categorized as *terrible* or *poor* in 14.9% of hysteroscopies and *moderate* , *good* , or *excellent* in 85.1% ( Figure 1 ).

A *terrible* or *poor* tolerance was more frequently reported in menopausal women (18.1% vs. 11.7% in pre-menopausal women, *p* = 0.001) and women with no previous vaginal delivery (18.8% vs. 12.9% in women with at least one vaginal birth, *p* = 0.007). Patients considered to have had *terrible* and *poor* tolerance were older than patients with *moderate* to *excellent* tolerance (55.2 ± 14.1 years vs. 52.8 ± 13.7 years, *p* = 0.019). Among patients with intracavitary pathology, those whose tolerance was evaluated as *moderate* , *good* , or *excellent* underwent operative hysteroscopy in 55.5% of cases, whereas only 21.8% of patients whose tolerance was rated as *terrible* or *poor* had an operative procedure ( *p* < 0.0005). Previous cesarean delivery, menopausal hormone therapy, tamoxifen hormone therapy, priming with misoprostol and paracervical block showed no statistically significant association to office hysteroscopy tolerance. Cervix/uterine cavity was inaccessible in 2.7% of patients. Inability to complete the procedure due to patient intolerance occurred in 1.7% of cases in which uterine cavity was accessed. Low tolerance led more often to scheduling a second hysteroscopic procedure under anesthesia (56.4% vs. 17.5% in *moderate* -to- *excellent* tolerance, *p* < 0.0005). Upon performing multivariate logistic regression analysis, menopausal status and previous vaginal delivery were shown to be the only variables significantly associated with procedure tolerance ( Table 2 ).

**Table 2:** Factors associated with tolerance to office hysteroscopy – logistic regression analysis

Variables	Odds ratio	*P* -value	95% confidence interval
Menopause	0.556	0.026	0.333–0.931
Previous vaginal delivery	1.922	0.000	1.34–2.758
Age	0.975	0.994	0.975–1.013

## Discussion

In this study, office hysteroscopy was well tolerated by 85% of patients, and adequate diagnostic hysteroscopy was possible in 95.7% of procedures. This is in accordance with data published by Bettocchi et al. 13 in an analysis of 31,052 rigid office hysteroscopies performed between 1996 and 2014 that reported complete evaluation of the uterine cavity in 94% of patients. Other published series displayed completion rates ranging from 77 to 97.2%, 14 with most cited reasons for failure being cervical stenosis, pain, vagal reaction, and anxiety. 2 In our study, cervical stenosis was the main factor hampering hysteroscopy outcome, and among the patients in which the intrauterine cavity was accessible, pain was the limiting factor, calling for procedure interruption in 1.7% of cases. In this study, menopause was one of the predictive factors for low tolerance to office hysteroscopy. Previous studies had already pointed out this association. Menopausal women have a narrow cervical canal due to atrophy, accounting for a painful passage of the hysteroscope through the cervix. 8 As these patients are the ones at a greater risk of intracavitary pathology, they are the group that would most benefit from complete and well tolerated hysteroscopic procedures. As such, providing the optimal conditions for reducing procedure pain and ensuring a successful hysteroscopy is particularly important in this population.

De Iaco et al. 15 suggested that pain scores during outpatient hysteroscopy only correlated with age and were not affected by menopausal status or parity. Our multivariate analysis did not show an association between age and procedure tolerance, probably because age is a confounder for menopause. Former vaginal delivery has been pointed out as a facilitating factor for hysteroscope passage through the internal cervical ostium. De Carvalho Schettini et al. 3 showed that previous vaginal deliveries reduced pain risk in 30% of cases. Our results corroborate this; however, no association with office hysteroscopy tolerance was found for nulliparity or previous cesarean delivery. Menopausal hormone deprivation favors cervical stenosis and uterine involution, so menopausal hormone therapy and antiestrogen therapy for breast cancer might have an impact on procedure pain 3 but we did not observe this, possibly due to a low number of cases, meaning less statistical power. Regarding cervical softening, priming with misoprostol has been shown to decrease outpatient hysteroscopy pain. 1 16 Issat et al. 17 emphasized that the effect does not depend on age, hormonal status, parity, or type of outpatient hysteroscopy. 17 Studies vary widely on dose, route of administration, and time before the procedure. 18 In our study, misoprostol was prescribed as a single sublingual dose of 400 micrograms, with no apparent impact on hysteroscopy tolerance.

Some studies have tried to understand the benefit of combining different measures for pain reduction. Previous results by Ghamry et al. 6 have shown that misoprostol plus local anesthesia appears to be the best pharmacological approach for pain reduction during hysteroscopy, but in our study, only 4 cases had simultaneous application of both misoprostol and local anesthesia. Paracervical anesthesia is particularly important in reducing pain associated with cervical manipulation during tenaculum placement. 6 Vaginoscopic approach is the current practice in our department for years and it obviates the need for speculum and tenaculum insertion. This might explain why paracervical block was not associated with improving tolerance scores in our study and the few number of cases in which it was applied. We observed that when office hysteroscopy was well tolerated, patients with intracavitary pathology were more likely to be successfully submitted to operative procedures, while low tolerance was associated with the need for a second hysteroscopy under anesthesia, increasing risks, recovery time, and costs. This study reflected the experience of a tertiary center with a systematic approach to hysteroscopy, which has been implemented for years. Finally, addressing our study limitations, we point out its retrospective nature. Our findings may not be generalizable to centers with less experience in a vaginoscopic approach. We did not take into account premedication with non-steroidal antiinflammatory drugs or supplemental vaginal estradiol, as many patients failed to report this to the operator and this information did not feature in their medical record. Additionally, our appraisal of patient tolerance to office hysteroscopy was based on subjective perception by the operator, and women's level of satisfaction was not taken into account.

## Conclusion

Office hysteroscopy is a generally well-tolerated procedure. Menopause and lack of previous vaginal delivery are associated with low tolerance to office hysteroscopy, but most women in these groups are able to go through the procedure successfully. However, low tolerance negatively impacts patient cooperation and makes it more likely for a second hysteroscopic procedure under general anesthesia to be necessary, with the entailed additional risks and costs. Considering that pain is the main reason for outpatient procedure failure, it becomes of the utmost interest to discern the factors associated with poor tolerability and to implement pain prevention measures and relief strategies as appropriate. Our work suggests that some groups of patients are more likely to benefit from pain relief measures during office hysteroscopy. It is important to screen these patients, anticipate their individual needs, tailoring to each one accordingly and ensuring a painless procedure.
